# Identification and Functional Analysis of circRNAs during Goat Follicular Development

**DOI:** 10.3390/ijms25147548

**Published:** 2024-07-09

**Authors:** Jie Liu, Conghui Guo, Junjie Fu, Dewu Liu, Guangbin Liu, Baoli Sun, Ming Deng, Yongqing Guo, Yaokun Li

**Affiliations:** 1 College of Animal Science, South China Agricultural University, Guangzhou 510642, China; liu17860720028@163.com (J.L.); guoconghui94@126.com (C.G.); baolisun@scau.edu.cn (B.S.); dengming@scau.edu.cn (M.D.); yongqing@scau.edu.cn (Y.G.); 2 National Local Joint Engineering Research Center of Livestock and Poultry, South China Agricultural University, Guangzhou 510642, China

**Keywords:** goat, circRNA, follicles, reproduction

## Abstract

Litter size is a crucial quantitative trait in animals, closely linked to follicular development. Circular RNA (circRNA), a type of single-stranded closed-loop endogenous RNA with stable expression, plays pivotal roles in various biological processes, yet its function in goat follicular development remains unclear. In this study, we collected large (follicle diameter > 3 mm) and small (1 mm < follicle diameter < 3 mm) follicles from black goats in the Chuanzhong region for circRNA sequencing, with the aim of elucidating the functional circRNAs that influence follicle development in goats. Differential analysis revealed that 17 circRNAs were upregulated in large follicles, and 28 circRNAs were upregulated in small follicles. Functional enrichment analysis revealed significant enrichment of pathways related to reproduction, including cellular response to follicle-stimulating hormone stimulus, the PI3K-Akt signaling pathway, the MAPK signaling pathway, and the Notch signaling pathway. Based on the ceRNA mechanism, 45 differentially expressed circRNAs were found to target and bind a total of 418 miRNAs, and an intercalation network including miR-324-3p (circRNA2497, circRNA5650), miR-202-5p (circRNA3333, circRNA5501), and miR-493-3p (circRNA4995, circRNA5508) was constructed. In addition, conservation analysis revealed that 2,239 circRNAs were conserved between goats and humans. Prediction of translation potential revealed that 154 circRNAs may potentially utilize both N6-methyladenosine (m6A) and internal ribosome entry site (IRES) translation mechanisms. Furthermore, the differential expression and circularization cleavage sites of five circRNAs were validated through RT-qPCR and DNA sequencing. Our study constructed a circRNA map in goat follicle development, offering a theoretical foundation for enhancing goat reproductive performance.

## 1. Introduction

Goat meat is highly favored for its delicious taste, richness in protein, and low fat content [1]. However, the lower fecundity of goats has significantly impeded the development of the goat industry. The ovary, comprising follicles as the basic unit, is a vital reproductive organ in female animals. It has been reported that in mammals, only 1% of follicles develop to a mature follicle and ovulation occurs, while the rest go on to an atretic fate [2].The ovulation rate is closely associated with fertility and represents a significant factor influencing litter size. Within a typical estrous cycle, mice exhibit ovulation of up to 19 eggs [3], while pigs can ovulate as many as 30 eggs [4]. In contrast, goats typically ovulate only 1–3 eggs [5], a factor that substantially diminishes the litter size in this species. Oogenesis is a complex biological process regulated by granulosa cells and membrane cells within the follicle, as well as by extrafollicular hormones [6]. High-quality oocytes are essential for the perpetuation of species, underscoring the importance of investigating the intrinsic mechanisms of follicular ovulation in goats for the sustainability of the goat industry.

Circular RNA (circRNA) is a single-stranded, closed RNA molecule lacking a 5′ cap and a 3′ polyA tail, imparting it with higher stability compared to linear RNA [7]. The discovery of circRNA traces back to its first observation in plants by Sanger in 1976 [8]. Subsequently, its presence has been documented across various organisms, including prokaryotes, eukaryotes, and mammals [9,10]. For a considerable duration, circRNAs were regarded as products of erroneous splicing. However, through extensive investigation, the functional repertoire of circRNAs has been substantially expanded. Primarily, they serve as miRNA sponges, interact with RNA-binding proteins, translate proteins, and engage with parental genes [11,12,13,14], thereby exerting distinctive regulatory influences across a diverse array of biological processes. It was found that circSLC41A1 resisted apoptosis in porcine follicular granulosa cells by targeting miR-9820-5p [15], circRALGPS2 could translate a 212-amino-acid protein to promote autophagy in chicken granulosa cells [16], and apoptosis in duck granulosa cells was promoted after overexpression of aplacirc_13267 [17]. This shows that circRNAs have an important role in animal reproduction; however, little research has been reported on circRNAs in goat reproduction.

The Chuanzhong black goat is an outstanding local meat breed from southern China. Through a lengthy process of natural selection and artificial breeding, it has developed several advantageous traits, including the ability to produce multiple lambs per litter, year-round estrus, rapid growth and development, and strong adaptability. Consequently, it holds a significant position among ruminants in southern China [18]. The lambing rate of first-time Chuanzhong ewes is 197%, while the lambing rate of mature Chuanzhong ewes is 249%. These rates clearly surpass those of other southern Chinese goat breeds, including Leizhou goats, making the Chuanzhong breed an invaluable resource for studying the reproductive performance of goats in southern China. However, compared to species like pigs, the relatively lower reproductive efficiency has long been a bottleneck in the development of the goat industry. Therefore, to enhance breeding efficiency in goats, it is imperative to delve into the regulatory mechanisms affecting litter size in goats.

Therefore, in this experiment, we collected large and small follicles during estrus from Chuanzhong black goats in China for circRNA sequencing, with the aim of investigating the functional circRNAs influencing follicular development in goats.

## 2. Results

### 2.1. Identification of Follicular circRNA

High-quality sequencing data are essential for subsequent analyses. Following quality control of the raw sequencing data, it was observed that the Q20 values for all samples exceeded 98%, the Q30 values were above 93%, and the percentage of valid reads was approximately 90% (Table 1). These findings indicate the high credibility of the sequencing data.

**Figure 1:**
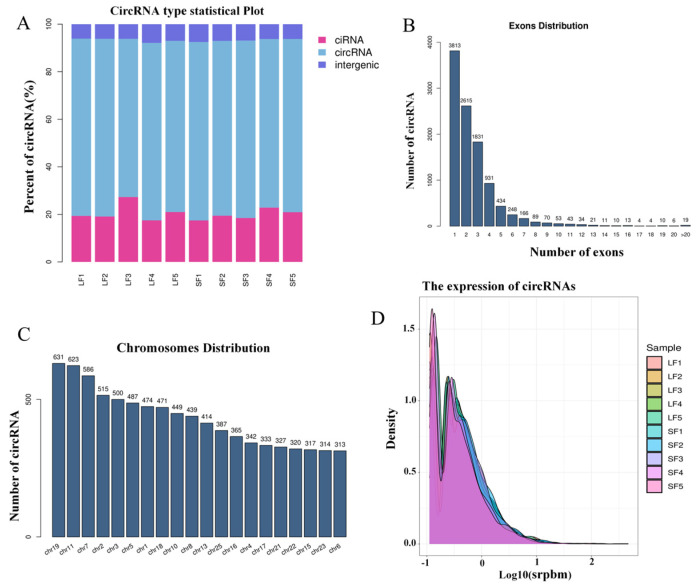
Identification of circRNA. (**A**) Types of circRNA. (**B**) Number of exons contained in circRNAs. (**C**) Chromosomal distribution of circRNA. (**D**) Expression statistics of circRNA.

The identification of circRNAs was based on their reverse splicing characteristics. In total, 10,425 circRNAs were identified across all samples in this study (Table S1), which were classified as intronic circRNAs, exonic circRNAs, and intergenic circRNAs. Exonic circRNAs accounted for more than 60% of each sample (Figure 1A), with most circRNAs consisting of 1–4 exons (Figure 1B). Moreover, the analysis of circRNA origin across chromosomes showed that chromosomes 19, 11, and 7 generated more circRNAs compared to other chromosomes (Figure 1C). Additionally, an analysis of circRNA expression revealed that the expression levels of circRNAs in goat follicles were generally low (Figure 1D).

### 2.2. Differential Expression Analysis of Follicular circRNAs

**Figure 2:**
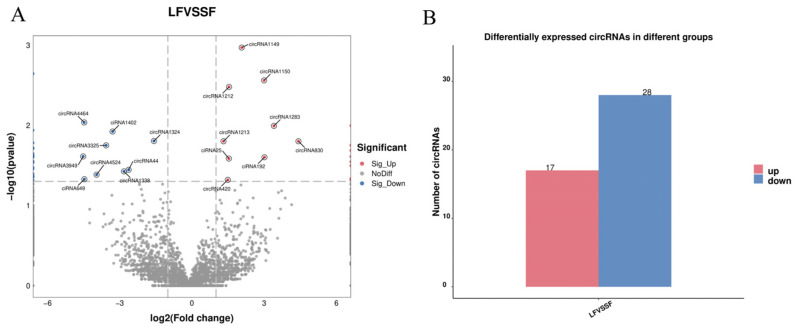
Differential circRNA analysis in small and large follicles. (**A**) Volcano map of differentially expressed circRNAs. (**B**) Bar graph of differentially expressed circRNAs.

Differential analysis of the circRNAs identified by sequencing based on expression revealed a total of 45 differentially expressed circRNAs between large and small follicles (Figure 2A). Of these, 17 circRNAs were upregulated in large follicles and 28 in small follicles (Figure 2B).

### 2.3. Functional Enrichment Analysis of Differential circRNA Parental Genes

**Figure 3:**
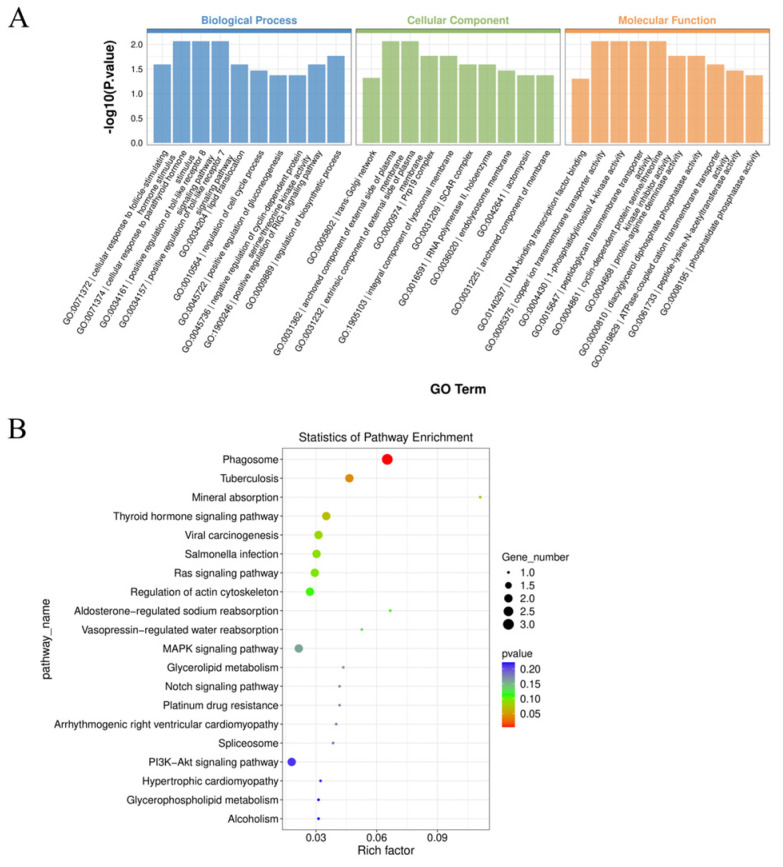
Enrichment analysis of differentially expressed circRNA parental genes. (**A**) GO enrichment analysis of circRNA parental genes. (**B**) KEGG enrichment analysis of circRNA parental genes.

Functional enrichment analysis of parental genes associated with circRNAs provides valuable insights into circRNA function. The parental genes of circRNAs exhibiting significant differences between small and large follicles were subjected to functional enrichment analysis. GO analysis revealed significant enrichment of 123 terms (Table S2), including processes such as cellular response to follicle-stimulating hormone stimulus, positive regulation of the RIG-I signaling pathway, regulation of the cell cycle process, and positive regulation of gluconeogenesis, among others (Figure 3A). KEGG analysis showed that important signaling pathways such as the PI3K-Akt signaling pathway, the MAPK signaling pathway, and the Notch signaling pathway were enriched (Figure 3B) (Table S3).

### 2.4. Interaction Network Analysis of Differential circRNAs with miRNAs

**Figure 4:**
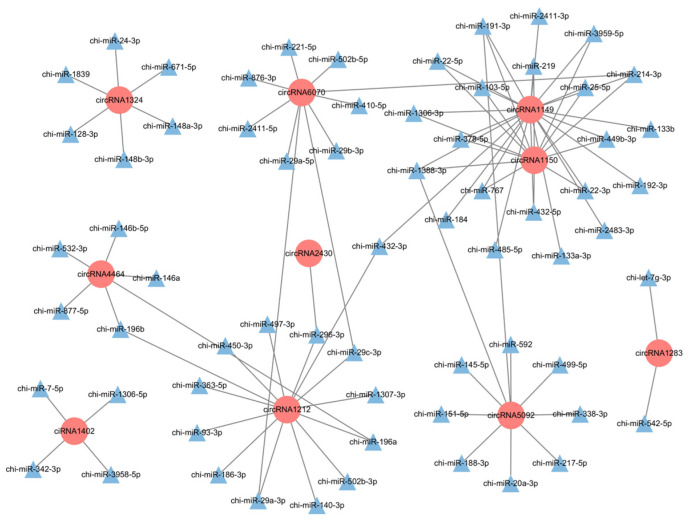
Interaction network of differentially expressed circRNAs with miRNAs.

circRNAs play a crucial regulatory role by acting as miRNA sponges. Using TargetScan (version 5.0) and miRanda (version 3.3a) predictions with intersecting results, we identified 45 differentially expressed circRNAs that target a total of 418 miRNAs (Table S4). Subsequently, we selected the top 10 circRNAs based on differential *p*-values to construct a network illustrating their interactions with miRNAs (Figure 4). Among these, miR-324-3p, miR-202-5p, miR-493-3p, miR-17-5p, miR-378-3p, miR-21-5p, and other potentially interacting miRNAs were identified.

### 2.5. Prediction of the Translational Potential of circRNA

**Figure 5:**
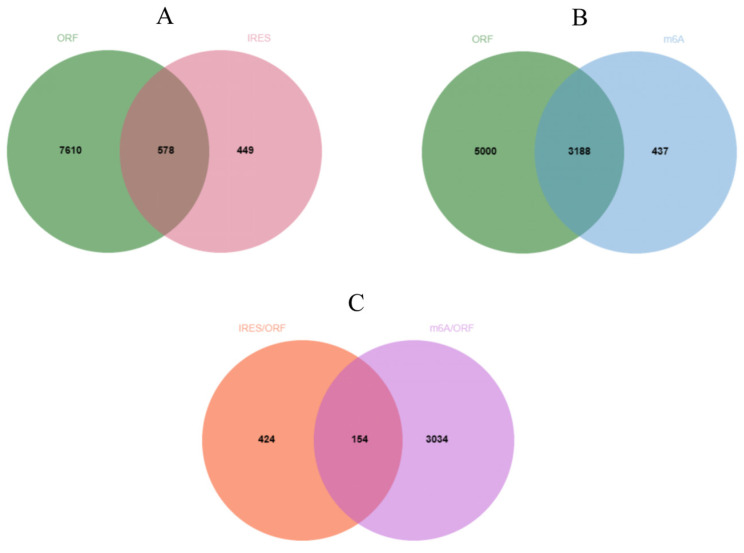
Interaction network of differentially expressed circRNAs with miRNAs. (**A**) CircRNAs simultaneously harboring ORFs and IRESs. (**B**) CircRNAs simultaneously harboring ORFs and m6A. (**C**) CircRNAs that rely on both IRESs and m6A to initiate translation.

CircRNAs have the potential to play significant biological roles through translation into short peptides. In this study, ORFfinder analysis revealed that 8188 circRNAs contained open reading frames (ORFs) spanning the splicing site. Additionally, IRESfinder prediction identified 1027 circRNAs harboring Internal Ribosome Entry Sites (IRESs), and upon intersecting with those possessing open reading frames, 578 circRNAs were identified as potentially capable of protein translation (Figure 5A) (Table S5). SRAMP analysis indicated that 3625 circRNAs exhibited high-confidence m6A modification sites, and subsequent intersection with circRNAs containing open reading frames revealed 3188 candidates with protein translation potential (Figure 5B) (Table S6). Furthermore, the intersection of circRNAs predicted by both methods unveiled 154 circRNAs with dual translational mechanisms (Figure 5C) (Table S7), which could be crucial for regulating follicle development in goats.

### 2.6. Conservativeness Analysis of circRNA

CircRNAs that exhibit conservation across species are of particular interest for study. Comparison of circRNAs identified through sequencing with human circRNAs from circBase indicated that a total of 2239 circRNAs were conserved between goats and humans (Table S8). Among these, seventeen circRNAs displayed significant differences and potentially exert similar biological effects in both humans and goats (Table 2).

### 2.7. RT-qPCR and DNA Sequencing Validation of Differential circRNAs

**Figure 6:**
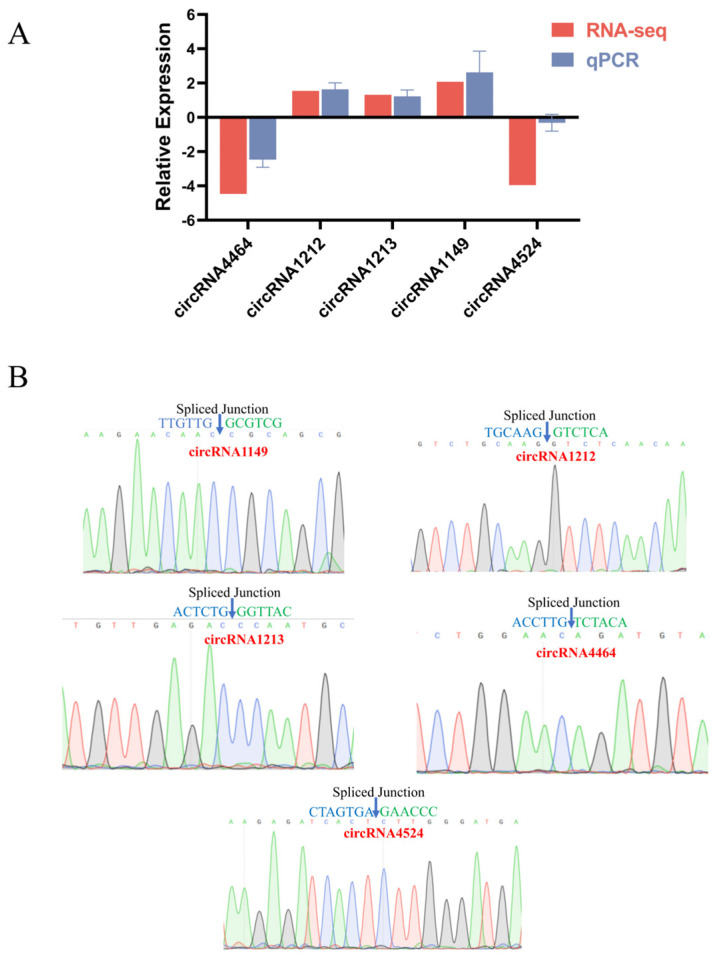
Validation of differentially expressed circRNA. (**A**) RT-qPCR verification of circRNA expression trend. (**B**) DNA sequencing validates the circularization shear site of circRNAs.

The accuracy of sequencing data is crucial for subsequent studies. To verify the expression of circRNAs in small and large follicles, five circRNAs were randomly selected for qPCR analysis, and the resulting products were subjected to Sanger sequencing. The obtained results demonstrated a consistent expression pattern with the sequencing data (Figure 6A), and the cyclization shear sites corresponded accurately with the sequencing sequences (Figure 6B). These findings indicate the reliability and accuracy of the sequencing data utilized in this study.

## 3. Discussion

Excellent reproductive performance in animals is essential for the continuation and expansion of the species. Key to producing a large number of oocytes is good follicular development. This development is a complex and sophisticated process influenced by communication between follicular granulosa cells, membrane cells, and oocytes. Therefore, investigating the molecular mechanisms of follicular development is crucial for analyzing reproductive performance [19]. The goat, as a crucial economic animal renowned for its delectable meat, faces challenges in reproductive efficiency that impede the advancement of the goat industry. Previous research has highlighted the significance of BMP6 [20], WNT4 [21], NMUR2 [22], miR-130a-3p [23], and miR-101-3p [24] in regulating proliferation, apoptosis, and hormone secretion in goat granulosa cells. However, circRNA, as an emerging star molecule, has been little reported on regarding follicle development in goats. Therefore, in this study, the small and large follicles of black goats from Chuanzhong were collected for circRNA sequencing to identify circRNAs involved in follicular development. The results demonstrated that each sample yielded over 10 G of effective data, sufficient for detecting transcripts with low expression [25].

A total of 10,425 circRNAs were detected across all samples, predominantly composed of exonic sequences, consistent with findings in Wuan goat muscle tissue [26], but differing from chicken follicular granulosa cells where intronic circRNAs are more prevalent [27], possibly due to species-specific and environmental factors. Prior research has indicated lower circRNA expression across species [28], a trend supported by our sequencing results. Analysis of circRNA origin revealed chromosome 19 as the primary source, contrasting with findings in chickens [29] and pigs [30]. In summary, our characterization of circRNAs in goat follicles highlights their species- and tissue-specific nature.

Differential analysis revealed 45 significantly different circRNAs, with 17 upregulated in large follicles and 28 in small follicles. To elucidate potential biological functions, functional enrichment analysis was performed on the parental genes of these circRNAs. GO analysis highlighted significant enrichment in the cellular response to follicle stimulating hormone. Follicle stimulating hormone, a pituitary gland-secreted gonadotropin, plays a crucial role in promoting follicular development and maturation, synergizing with luteinizing hormone to regulate steroid hormones and physiological activities in animals [31]. Within this pathway, EFNA5, a member of the receptor tyrosine kinase family (RTKs), exhibited significant association. Studies in mice have shown EFNA5’s necessity in responding to gonadotropins and completing ovulation, with EFNA5 deficiency resulting in reduced fertility [32]. Additionally, in sheep, follicle stimulating hormone promotes EFNA5 expression in follicles, implicating EFNA5 in animal follicular development [33]. Notably, circRNA4464, originating from EFNA5, displayed significant differences between large and small follicles, upregulating in small follicles, possibly inhibiting follicular cell response to follicle stimulating hormone, thereby impeding follicular development. Furthermore, RPRD1B, a transcriptional co-activator, was associated with the significantly enriched regulatory cell cycle pathway. RPRD1B encodes a nuclear protein that binds to Aurora B, regulating cell cycle protein CCNB1 expression, accelerating G2 to S phase transition in gastric cancer cells [34]. Similarly, in chicken DF-1 cells, RPRD1B overexpression significantly promoted G1 to S phase cell cycle transition [35]. Based on sequencing data, significantly different circRNA44, derived from reverse shearing of RPRD1B, was observed in the small and large follicles of Kawanaka goats, suggesting circRNA44’s potential role in follicular cell cycle regulation. The KEGG analysis revealed enrichment of several signaling pathways associated with reproduction, including the MAPK, PI3K-Akt, and Notch pathways. Previous studies have demonstrated the impact of these pathways on various aspects of follicular development. For example, enhancing the MAPK signaling pathway promotes goat oocyte maturation [36], while activating the PI3K-AKT pathway stimulates the proliferation of follicular granulosa cells [37] and enhances estradiol secretion in goat follicular membrane cells [38]. Additionally, inhibition of the Notch signaling pathway has been linked to a decrease in the number of primordial follicles in mice [39]. Furthermore, analysis of sequencing data revealed significant associations between the parental genes of circRNA4464, circRNA830, circRNA1212, circRNA1213, circRNA4464, and circRNA6035 with these pathways. This suggests that these circRNAs may play crucial roles in follicular development in goats, although further experiments are warranted to elucidate their specific biological functions.

The sponge mechanism has become a hotspot of circRNA research. In this study, we predicted that 45 differentially expressed circRNAs targeted and bound a total of 418 miRNAs, including miR-324-3p, miR-202-5p, miR-493-3p, miR-17-5p, miR-378-3p, and miR-21-5p. Studies have demonstrated that miR-324-3p suppresses the proliferation of goat follicular granulosa cells by targeting DENND1A [40]; miR-202-5p downregulates BCL2 expression, thereby inducing apoptosis in goat granulosa cells [41]; overexpression of miR-493-3p leads to decreased JAK3 expression, resulting in the inhibition of goat follicular granulosa cell proliferation and reduced secretion of E2 [42]. In addition, Zhang et al. discovered that miR-17-5p suppressed proliferation and estradiol synthesis in porcine follicular granulosa cells [43]. Sun et al. demonstrated that miR-378-3p preserves the quantity of primordial follicles in mice via autophagy [44]. Xue et al. revealed that miR-21-5p enhances glucose uptake in porcine granulosa cells [45]. Based on biosignature prediction, miR-324-3p (circRNA5501, circRNA5650, ciRNA192, circRNA2497, circRNA3333), miR-202-5p (circRNA3333, circRNA5501), miR-493-3p (circRNA3333, circRNA4892, circRNA4995, circRNA5501, circRNA5508), miR-17-5p (circRNA5501, circRNA1405, circRNA3325, circRNA3333, circRNA5501), miR-378-3p (circRNA2497, circRNA5501, circRNA5508, circRNA5650), and miR-21-5p (circRNA5501) exhibit potential targeting relationships. Therefore, we speculate that these circRNAs may play indispensable roles in goat follicular development. Furthermore, it is evident from interactions that a single circRNA may target multiple miRNAs, indicating the complexity of circRNA function.

As research progresses, the translational capacity of circular RNAs (circRNAs) has been increasingly recognized, with internal ribosome entry site (IRES)-mediated translation being the primary mechanism. By intersecting predictions from ORFfinder and IRESfinder, 578 circRNAs were identified as potential candidates for protein translation. Notably, examples such as circ-EIF6, which promotes breast carcinogenesis through the encoding of a 224 amino acid polypeptide [46], and circNEB, which enhances proliferation and differentiation of bovine skeletal muscle via a 907 amino acid polypeptide [47], underscore the translational potential of circRNAs. While there is currently limited literature on the translation of proteins by circRNAs, this insight suggests that circRNAs may translate peptides, thereby exerting unforeseen effects on goat follicular development, thus offering novel avenues for regulating follicular development in goats.

Conservation of genes across species can facilitate cross-species migration studies, and conserved circRNAs may also play significant roles. In this study, a total of 2239 circRNAs were found to be conserved to some extent with humans. Among them, the upregulation of hsa_circ_0008193 inhibits the proliferation and migration of lung pancreatic cells [48], while the downregulation of hsa_circ_0004458 inhibits the proliferation of thyroid cancer cells [49]. Moreover, hsa_circ_0023409 promotes the progression of gastric cancer cells by activating the IRS4/PI3K/AKT pathway, thus facilitating gastric cancer cell progression [50]. These conserved circRNAs provide valuable insights for research on goats and offer data support for utilizing human circRNA databases in goat studies, thereby facilitating circRNA research in goats.

## 4. Materials and Methods

### 4.1. Sample Collection

**Figure 7:**
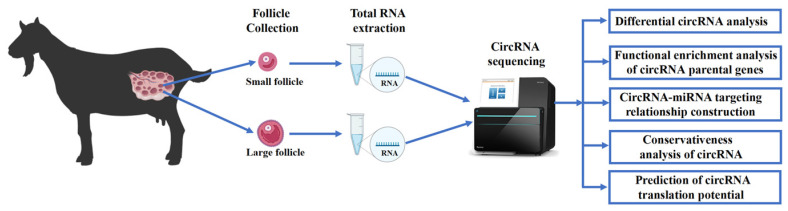
Overall flowchart of the pilot study.

Chuanzhong black goats sourced from the experimental farm of South China Agricultural University were selected as the test subjects. All experimental goats, aged between 2 and 3 years old, had undergone 2 to 3 lactation cycles, with an average weight of 35.31 ± 1.71 kg. The goats were uniformly housed in a standardized environment consisting of high-bed, wooden leaky-floor enclosures, and were provided with ad libitum access to feed and water. All experimental goats underwent hormonal treatment prior to follicle collection: CIDR embedded bolus was implanted on day 0, and prostaglandin was injected and withdrawn on day 14. Euthanasia was conducted two days post-treatment, with both ovaries collected thereafter. Follicles of all sizes were subsequently isolated under somatoscopy (large follicle diameter > 6 mm, 1 mm < small follicle diameter < 3 mm) and promptly stored in liquid nitrogen. Total RNA was extracted and subjected to circRNA sequencing and analysis (Figure 7).

### 4.2. Total RNA Extraction, Library Construction and Sequencing

Total RNA was extracted from follicles of black goats in Chuanzhong using Trizol reagent (Invitrogen, Carlsbad, CA, USA) following the manufacturer’s instructions. The quality and quantity of the extracted RNA were assessed using a Bioanalyzer 2100 with the RNA 6000 Nano LabChip Kit (Agilent, Santa Clara, CA, USA), ensuring RNA integrity number (RIN) values greater than 7. RNA samples exceeding 5 μg were subjected to ribosomal RNA (rRNA) depletion using the Ribo-Zero Gold rRNA Removal Kit (Illumina, San Diego, CA, USA). Following rRNA depletion, the RNA was treated with RNase R to enrich circular RNAs by incubation at 37 °C for 30 min. The enriched RNA was fragmented using divalent cations under elevated temperatures.

To prepare for cDNA synthesis, the fragmented RNA underwent reverse transcription using SuperScript™ II reverse transcriptase (Invitrogen, Waltham, MA, USA). Subsequently, second-strand DNA was synthesized using *E. coli* DNA polymerase I, RNase H, and dUTP Solution. Adenine bases were added to the blunt ends of the synthesized DNA fragments for subsequent ligation to indexed adapters containing thymine bases. Finally, the cDNA fragments were size-selected (300–600 bp) using AMPure XP beads (Beckman Coulter, Brea, CA, USA) and subjected to PCR amplification to generate a cDNA library with a final size of 300 ± 50 bp.

The constructed cDNA library was sequenced using paired-end sequencing on an Illumina NovaSeq™ 6000 platform following standard protocols.

### 4.3. Analysis of circRNA Sequencing Data

To ensure high-quality sequencing data, adapters and low-quality bases were filtered out from paired-end sequencing data using Cutadapt. All subsequent analyses were conducted based on these high-quality clean reads. Reads from all samples were aligned to the goat reference genome using Tophat2 (version 2.0.4), and circRNAs were identified using CIRCExplorer2 and CIRI.

### 4.4. Differential Expression Analysis of circRNA

Differential expression analysis of circRNAs from small and large follicles was performed using edgeR (http://bioconductor.org/packages/release/bioc/html/edgeR.html) (accessed on 30 December 2023). *p* value < 0.05, fold change > 2 and fold change < 0.5 were considered as differentially expressed circRNAs.

### 4.5. GO and KEGG Analysis of Differential circRNA Host Genes

To elucidate potential biological functions associated with differential circular RNAs, enrichment analyses of parental genes for differentially expressed circRNAs were conducted using Gene Ontology (GO) and the Kyoto Encyclopedia of Genes and Genomes (KEGG). A significance threshold of *p* < 0.05 was applied to identify pathways showing significant enrichment.

### 4.6. Prediction of Target miRNAs for circRNAs

TargetScan (version 5.0) and miRanda (version 3.3a) were employed for the prediction of potential miRNAs targeted for binding. Since raw prediction might contain false positives, the results from both software were combined to identify miRNAs likely to be targeted. Thresholds of TargetScan_Score ≥ 50 and miranda_Energy < −10 were applied for screening the candidate miRNAs.

### 4.7. Prediction of the Translational Potential of circRNA

Translation potential exists for circRNAs, with internal ribosome entry sites (IRESs) serving as an important regulatory element for circRNAs to undergo translation in the absence of a 5′ cap structure. Due to the phenomenon of rolling-circle translation in circRNAs, each circRNA sequence was duplicated. Subsequently, the open reading frame (ORF) [51] potentially spanning the splicing site was predicted using ORFfinder (https://www.ncbi.nlm.nih.gov/orffinder/) (accessed on 30 December 2023), while IRESfinder (https://github.com/xiaofengsong/IRESfinder) (accessed on 30 December 2023) was employed to identify internal ribosome entry sites (IRESs) [52]. Finally, the intersection of these two results was used to screen for circRNAs with translation potential.

### 4.8. Conservativeness Analysis of circRNA

CircRNAs exhibit a degree of conservation across species, and those conserved across species may serve significant functions. The approximately 200 bp sequences of goat circRNAs spanning the splice sites were analyzed for conservation with human circRNAs from circBase, employing the megablast algorithm (threshold evalue is 1 × 10^−10^). For circRNAs larger than 200 nt, the terminal 100 nt and the anterior 100 nt were utilized to generate a junction sequence. For those smaller than 200 nt, the posterior half of the sequence was relocated to the anterior end to create a junction sequence. CircRNAs whose comparison region spanned the shear site on both goat and human sequences were defined as being conserved.

### 4.9. RT-qPCR and DNA Sequencing Validation

To verify the accuracy of the sequencing data, five circRNAs were randomly selected for RT-qPCR and DNA sequencing verification. First, total RNA from follicles was reverse transcribed using the Evo M-MLV Reverse Transcription Premix Kit (Accurate Biology, Guangzhou, China) according to the manufacturer’s instructions. Subsequently, the expression was verified using 2× Ultra SYBR Green qPCR Mix (CISTRO, Guangzhou, China). The reaction system included 1 μL of cDNA, 0.5 μL of upstream primer, 0.5 μL of downstream primer, 10 μL of 2× Ultra SYBR Green qPCR Mix, and 8 μL of ddH_2_O. The thermal cycling conditions were 95 °C for 10 min, followed by 40 cycles of 95 °C for 5 s and 60 °C for 20 s. Finally, the qPCR products were subjected to Sanger sequencing to verify the base sequences of circRNA circularization shear sites.

## 5. Conclusions

In conclusion, we established a circRNA expression profile for goat ovarian follicles of various sizes and identified 45 circRNAs with significant differential expression. Functional enrichment analyses indicated that the MAPK signaling pathway, the PI3K-Akt signaling pathway, and the cellular response to follicle-stimulating hormone may play crucial roles in follicle development.

Reproduction-related interactions including miR-324-3p (circRNA2497, circRNA5650), miR-202-5p (circRNA3333, circRNA5501), and miR-493-3p (circRNA4995, circRNA5508) were constructed based on the ceRNA machinery network. Furthermore, 2239 circRNAs were found to be conserved with humans, while 578 circRNAs showed potential for protein translation. These findings provide valuable insights into the role of circRNAs in goat follicular development and offer a theoretical basis for enhancing goat fertility.

## Figures and Tables

**Table 1:** Sample data quality control statistics.

Sample	Raw Data Read	Valid Data Read	Valid Ratio (Reads)	Q20%	Q30%
LF1	92957118	83771828	90.12	99.20	95.20
LF2	94341552	84923578	90.02	99.09	95.16
LF3	94068076	84851900	90.20	99.19	95.35
LF4	82973554	76122068	91.74	99.58	95.33
LF5	75735446	67783970	89.50	98.81	93.54
SF1	93375512	84413204	90.40	99.12	95.34
SF2	90519034	81379192	89.90	99.19	95.36
SF3	77917452	70803598	90.87	99.64	95.89
SF4	80346710	71427864	88.90	98.78	93.69
SF5	90400602	82115126	90.83	99.18	94.48

Sample: sample name; Raw Data Read: the total number of reads in the downlinked data; Valid Data Read: the number of valid reads after removing splices and low-quality reads; Valid Ratio (reads): the percentage of valid reads; Q20%: the percentage of bases with a quality value ≥20 (a sequencing error rate <0.01); Q30%: the percentage of bases with a quality value ≥30 (a sequencing error rate <0.001) Q20%; the proportion of bases with a quality value ≥20 (a sequencing error rate less than 0.01); Q30%: the proportion of bases with a quality value ≥30 (a sequencing error rate less than 0.001).

**Table 2:** Conservativeness analysis of differentially expressed circRNAs.

Accession	Parent Gene	Conservative_circRNA/Parent Gene
circRNA1150	SERPINE2	hsa_circ_0005773/SERPINE2
circRNA1212	ITGB5	hsa_circ_0067080/ITGB5
circRNA5092	UBP1	hsa_circ_0007147/UBP1
circRNA1324	FAM120A	hsa_circ_0008193/FAM120A
circRNA830	MEF2C	hsa_circ_0129926/MEF2C
circRNA5493	ZNF536	hsa_circ_0109365/ZNF536
circRNA5508	intergenic_circRNA	hsa_circ_0004366/VPS13B
circRNA6643	PUM2	hsa_circ_0052867/PUM2
circRNA5501	intergenic_circRNA	hsa_circ_0064555/SATB1
circRNA5025	PLPP4	hsa_circ_0020207/PPAPDC1A
circRNA5671	MED13L	hsa_circ_0003059/MED13L
circRNA44	RPRD1B	hsa_circ_0007365/RPRD1B
circRNA1338	HSDL2	hsa_circ_0088087/HSDL2
circRNA5240	R3HDM2	hsa_circ_0002539/R3HDM2
circRNA420	RAB5C	hsa_circ_0106851/RAB5C
circRNA5490	ATP9B	hsa_circ_0108910/ATP9B
circRNA1922	LASP1	hsa_circ_0043388/LASP1

## Data Availability

The raw sequence data reported in this paper have been deposited in the Genome Sequence Archive (Genomics, Proteomics & Bioinformatics 2021) of the National Genomics Data Center (Nucleic Acids Res 2022), China National Center for Bioinformation/Beijing Institute of Genomics, Chinese Academy of Sciences (GSA: CRA015640) that are publicly accessible at https://ngdc.cncb.ac.cn/gsa (accessed on 30 March 2024).

## Supplementary Materials

The following supporting information can be downloaded at: https://www.mdpi.com/article/10.3390/ijms25147548/s1.
